# Skeletal Muscle Involvement in B-Cell Lymphoma: Two Cases Illustrating the Contribution of Imaging to a Clinically Unsuspected Diagnosis

**DOI:** 10.1155/2017/2068957

**Published:** 2017-04-30

**Authors:** Elijah Burton, Kristian Schafernak, Elaine Morgan, Jonathan Samet

**Affiliations:** ^1^David Grant USAF Medical Center, Fairfield, CA, USA; ^2^University of Arizona College of Medicine, Phoenix, AZ, USA; ^3^Mayo Clinic College of Medicine, Phoenix, AZ, USA; ^4^Northwestern University Feinberg School of Medicine, Chicago, IL, USA

## Abstract

Skeletal muscle lymphoma is rare, comprising only a very small subset of lymphoma cases. There are characteristic imaging features which, if recognized, can prevent delay in diagnosis and treatment, particularly when not suspected clinically. Herein, we report two cases of skeletal muscle lymphoma with nearly identical imaging features; the first is an example of primary muscle lymphoma in a 17-year-old boy with back and thigh pain, and the second represents lymphoma recurrence in a 55-year-old man with HIV. Characteristic features seen on MRI were key in raising suspicion for the disease and helped prevent a delay in pathologic diagnosis.

## 1. Introduction

Extranodal involvement is relatively common in NHL (non-Hodgkin Lymphoma), present in up to 30% of cases [1]. Involvement of the muscle, however, is much less frequent, representing 1.5–5% of extranodal lymphoma cases [2, 3]. Most of these cases showed contiguous spread from adjacent bone or other tissues, indicating that the musculature was not the primary origin of disease. In a study of 7000 patients with malignant lymphoma, primary muscle lymphoma was seen in only 8 patients [4]. By comparison, lymphoma of bone represents 5% of extranodal lymphomas and 7% of bone malignancies [4].

We report two cases of lymphoma presenting in muscle with progressive swelling and pain, one in a 17-year-old boy, and the other in a 55-year-old man with HIV. We aim to highlight the characteristic imaging findings of muscle lymphoma and to emphasize the importance of suggesting lymphoma as pathologic evaluation requires specific biopsy considerations.

## 2. Case Reports

### 2.1. Case 1

This patient is a 17-year-old boy who presented with a four-week history of intermittent back and thigh pain refractory to conservative therapy. The pain had progressively worsened, awakening him at night, and there was swelling in the thigh and buttocks. He denied fevers, night sweats, or weight loss at presentation. His past medical history was otherwise unremarkable and he had no history of trauma to the involved area. His exam and labs revealed that he was HIV-negative and afebrile without leukocytosis. Neurologic exam of the lower extremities showed no weakness or sensory deficit. After failing initial therapy with anti-inflammatory medications, his pediatrician sent him for an outpatient MRI. However, the pain was so severe that he was referred to the emergency department for adequate pain control prior to imaging.

**Figure 1:**
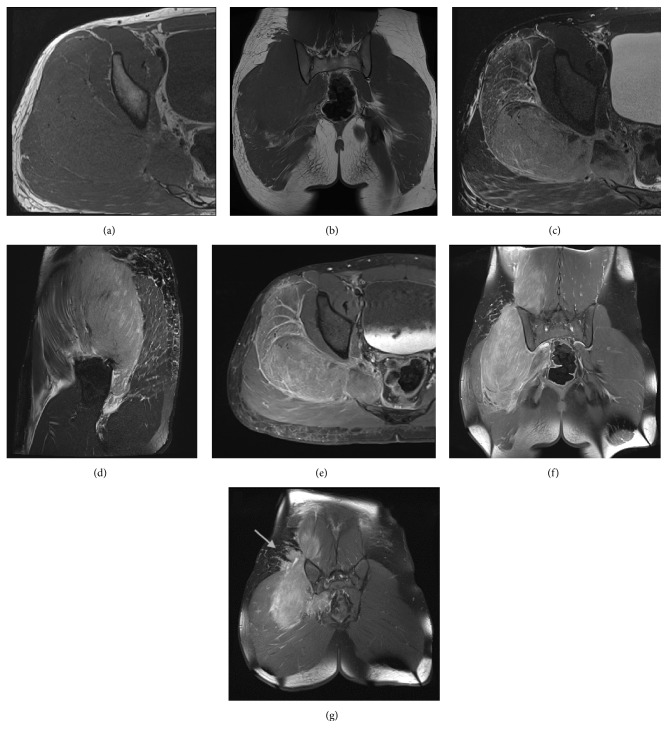
Axial T1 (a), coronal T1 (b), axial T2 (c), sagittal T2 (d), axial T1 FS postcontrast (e), coronal T1 FS postcontrast (f), and coronal T1 FS postcontrast (g) images of the involved lumbar spine and pelvis in the 17-year-old boy from case 1. There is unilateral marked expansion of the right gluteal, piriformis, and paraspinal muscles, exhibiting T1 isointensity and T2 hyperintensity. The muscle enlargement results in effacement of intramuscular fat. The postcontrast images demonstrate diffuse striated enhancement, giving an infiltrative appearance. Enhancing nodules are seen in the adjacent subcutaneous tissues. There is also subcutaneous fat stranding (indicated by the arrow, image (g)). Aside for the enlargement, note the overall preservation of muscle morphology and lack of a focal mass.

MRI demonstrated diffuse unilateral expansion of the right-sided paraspinal and gluteal musculature without a focal measurable mass. There was striking effacement of the normally identified fatty striations within the affected muscles. Relative to other muscles, the signal was isointense on T1. The muscle was diffusely mildly T2 hyperintense, with more dominant linear T2 hyperintense signal in a striated pattern along the muscle fibers. Following administration of gadolinium, the involved muscles exhibited diffuse enhancement with additional linear hyperenhancement in a striated pattern. Additionally, small enhancing soft tissue nodules adjacent to the affected muscles were seen in the overlying subcutaneous tissues (Figures 1(a)–1(g)). Although small, the presence of soft tissue masses was a clue pointing toward a neoplastic process and would be atypical for inflammatory or infectious myositis. These nodules were located over the buttocks, an unusual site for lymph nodes. Regardless, infectious myositis was also considered highly on the radiologic differential.

**Figure 2:**
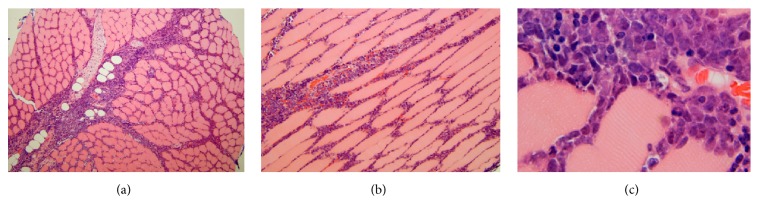
Transverse (a) and longitudinal (b) hematoxylin and eosin-stained histologic sections showing an infiltrate of intermediate-sized to large lymphoid cells in the endomysium between muscle fibers and the perimysium investing the fascicles or bundles of these fibers; these slides were prepared from the gluteal muscle biopsy of the 17-year-old boy described in case 1. On high magnification (c) one can observe the intimate association of the lymphoma cells with the individual striated muscle fibers.

**Figure 3:**
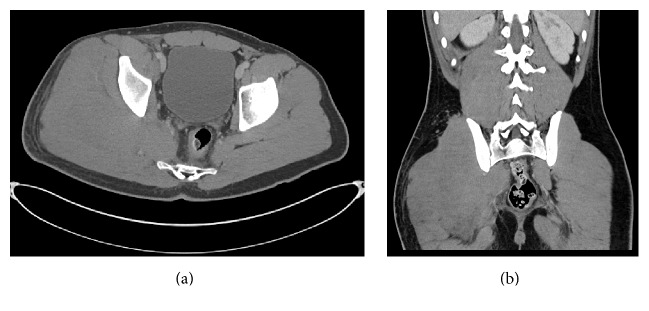
Axial (a) and coronal contrast enhanced CT (b) images of the abdomen and pelvis from the 17-year-old boy in case 1. There is striking asymmetric enlargement of the right gluteal and paraspinal muscles, isodense to adjacent muscle.

This appearance was suspicious for an infiltrative process, specifically lymphoma, which promptly led to ultrasound-guided biopsy of the involved gluteal musculature. A high-grade CD10 positive B-cell lymphoma was diagnosed (it was CD45 positive, CD19 positive, CD20 negative, CD10 positive, surface immunoglobulin negative, and BCL2 negative, with a Ki67 proliferation index approaching 100% based on limited phenotyping by combined flow cytometry and immunohistochemistry), but additional fresh tissue via open biopsy was requested for additional studies to attempt to refine the diagnosis. Ultimately, the lymphoma is perhaps best classified as a high-grade B-cell lymphoma, not otherwise specified, following the 2016 revision of the World Health Organization classification of lymphoid neoplasms (histopathology depicted in Figures 2(a)–2(c)). CT scan of the chest, abdomen, and pelvis was obtained for staging purposes and similar to the MRI, there was diffuse unilateral enlargement of the gluteal and paraspinal muscles (Figures 3(a) and 3(b)). There were a few suspicious local subcentimeter lymph nodes adjacent to the right psoas muscle enlargement. No other sites of involvement or additional lymph node enlargement were seen on the CT. Bone marrow biopsy and lumbar puncture were negative for involvement by lymphoma.

### 2.2. Case 2

This patient is a 55-year-old HIV-positive man who presented with several weeks of worsening pain and swelling in the lower leg. His past medical history was significant for CD10 positive diffuse large B-cell lymphoma of the kidney without bone marrow involvement, 15 months prior to presentation. He went into initial remission with chemotherapy. On exam, the patient was afebrile but his calf was exquisitely tender and enlarged. There was no neurologic deficit. After these symptoms progressed despite conservative therapy, MRI was ordered.

**Figure 4:**
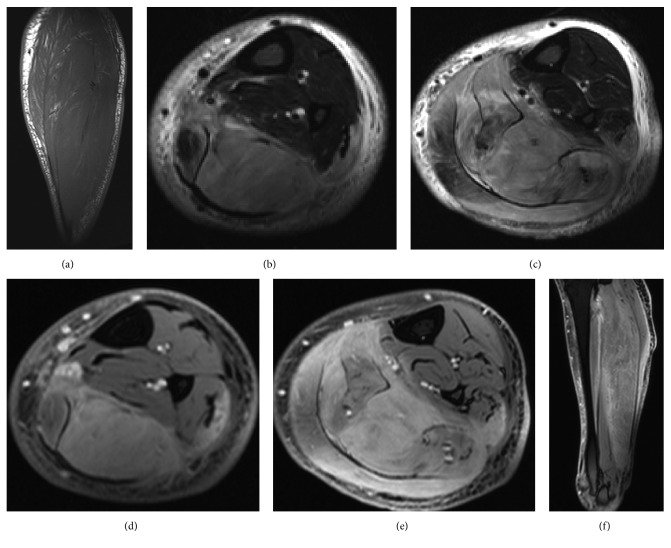
Sagittal T1 (a), axial STIR (b, c), axial T1 fat saturated postcontrast (d, e), and sagittal T1 fat saturated postcontrast (f) images of the involved lower leg in the 55-year-old man with HIV described in case 2. There is marked enlargement of the posterior calf muscles with effacement of the intramuscular fat. The affected muscle demonstrates T2 hyperintense signal and diffuse enhancement on postcontrast sequences. The overall findings should raise the suspicion for an infiltrative process such as lymphoma.

Similar to case 1, MRI demonstrated diffuse enlargement of multiple posterior calf muscles, with effacement of intramuscular fat (Figures 4(a)–4(f)). Postcontrast images showed diffuse enhancement of the affected muscles, but no focal mass (Figures 4(d)–4(f)).

**Figure 5:**
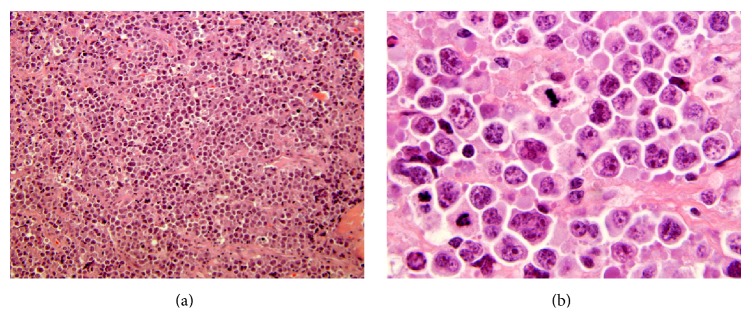
The 55-year-old patient's lymphoma from case 2 showed microscopically a more diffuse infiltrate of large cells with abundant mitotic figures (a, b).

**Figure 6:**
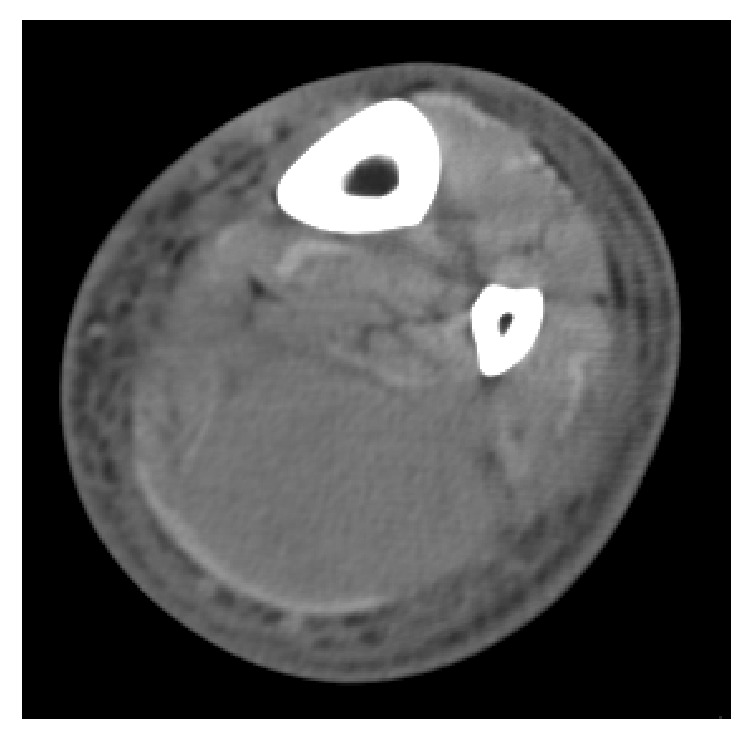
Axial contrast enhanced CT image of the 55-year-old man from case 2 showing diffuse enlargement of the soleus muscle which is homogenously iso- to slightly hypodense relative to adjacent muscle. Note the skin thickening and subcutaneous fat stranding, which can also be seen in lymphoma.

Myositis was favored over recurrent lymphoma clinically and radiologically. However, due to persistent symptoms, an open biopsy was performed, which revealed diffuse large B-cell lymphoma of the gastrocnemius muscle; histology is demonstrated in Figures 5(a) and 5(b). CT was subsequently ordered to assess for progression of disease, which redemonstrated diffuse enlargement of the posterior calf musculature and subcutaneous fat stranding of the leg (Figure 6). There was no lymph node enlargement in the chest, abdomen or pelvis, indicating that the muscle was the primary site of recurrence. Bone marrow biopsy and lumbar puncture were also negative for malignancy.

## 3. Discussion 

NHL commonly presents as extranodal disease. Although the definition of primary extranodal lymphoma is somewhat controversial, the commonly accepted definition is involvement of an organ with no or minor local lymph node enlargement [5]. Infiltration of the muscle is a very uncommon manifestation of lymphoma and most commonly occurs in the gluteal and pelvic musculature as a result of hematogenous dissemination or direct spread from adjacent lymph nodes or bone; primary involvement of the muscle is exceedingly rare [6]. By 1997, fewer than 50 cases of primary muscle lymphoma had been described [7]. Primary muscle lymphoma was found to account for just 0.1% of over 7,000 cases of lymphoma diagnosed over a 10-year period at the Mayo Clinic [8]. By comparison, in a study of 1168 patients with NHL, <1% involved the ovary and 3% involved bone [5].

Primary skeletal muscle NHL most commonly has an aggressive B-cell immunophenotype, but aggressive mature T-cell lymphoma has also been rarely reported [9]. It typically presents with a mass, swelling, and pain [10]. As imaging is often part of the initial workup, radiologists have the opportunity to direct further evaluation by suggesting the diagnosis.

Knowledge of the imaging findings associated with primary muscle lymphoma is limited to a small number of case reports and retrospective studies. Our patients' imaging findings correspond well with those described in the literature [7, 8, 10–13]. In a recent retrospective study, Chun et al. [14] assessed MRI findings from 20 biopsy-proven cases of muscle lymphoma. All patients had diffuse enlargement of the muscle. Nine of these were primary muscle lymphoma and 11 represented recurrence in the muscle. On T1-weighted images, all patients had equal to or slightly higher signal than normal surrounding muscle. All lesions demonstrated intermediate signal on T2-weighted imaging. In cases using contrast, the most common pattern was diffuse enhancement (seen in 13 of 19 cases); peripheral enhancement and marginal septal enhancement were the enhancement patterns seen in the remaining cases. A majority of patients showed stranding in the subcutaneous fat, with a minority demonstrating overlying skin thickening. Suresh et al. found that muscle lymphoma had a propensity to involve multiple muscle compartments, distinguishing it from most soft tissue sarcomas. Extension of muscle lymphoma into adjacent subcutaneous tissues was another interesting feature they reported and was seen in our first case presented [15]. Lymphoma can sometimes present with a focal muscle mass as opposed to an infiltrative ill-defined abnormality, but this is less common [16].

On CT, nearly all reports describe diffuse expansion of the muscle with iso- to hypodensity of the involved areas [2, 17–20]. There are two reported cases of NHL with extranodal muscle involvement which showed hyperdensity on CT. Both of these cases occurred in HIV-positive patients [21]. Our patient with HIV demonstrated iso- to hypodense muscle infiltration.

PET/CT was not performed in either of these cases and is not typically performed in the initial workup of muscle lymphoma. PET/CT is, however, often performed after a tissue diagnosis is made to obtain a baseline for which future scans can be compared to gauge response to therapy as well as for staging purposes [22]. PET/CT was not performed on the 17-year-old boy in the first case due to the severity of his disease at presentation and desire to initiate prompt therapy.

The differential diagnosis includes denervation muscle edema, infectious and inflammatory myositis, sarcoidosis, rhabdomyolysis, sarcoma, and metastatic disease, among other etiologies. Denervation edema of skeletal muscle can result in T2 hyperintensity and muscle expansion but may also present with weakness and evidence of nerve compression on imaging [23]. Inflammatory myositis such as polymyositis and juvenile dermatomyositis can display T2 hyperintensity in skeletal muscle but is rarely unilateral and can typically be excluded on the basis of normal laboratory values [24]. Muscle involvement of sarcoidosis on MRI shows multiple elongated nodules with a characteristic low signal center, producing a striped appearance. In addition, patients will typically have other manifestations of this systemic disease [25]. Rhabdomyolysis shows diffuse T2 hyperintense signal in a muscle and can be unilateral or bilateral. Postcontrast imaging can show diffuse enhancement or rim enhancement depending on the severity and time course. Laboratory tests and predisposing underlying conditions would differentiate this entity from lymphoma [26]. On MRI, primary soft tissue sarcomas typically present with a distinct rounded mass [27]. Intramuscular metastases appear as enhancing masses with central necrosis and peritumoral edema [28]. The absence of clinical and laboratory parameters of the aforementioned differential diagnoses should allow one to suggest lymphoma as the leading diagnosis.

Patients with HIV have a 60-fold greater risk of developing NHL compared to the general population. This risk is mainly present in individuals with CD4+ counts less than 200/mm^3^. There is a propensity for the development of extranodal disease in these patients as well, including primary or metastatic muscular involvement [29, 30]. When considering muscle lymphoma in an HIV-positive patient, infectious myositis should also be kept in mind. With this type of infection, most patients present with severe pain, fever, and muscle swelling. In its early stages, MRI will show muscle enlargement and T2 hyperintensity. In more progressed cases, MRI demonstrates intramuscular fluid collections which can represent abscesses or myonecrosis [31]. Nonbacterial myositis can have a wide spectrum of imaging findings depending on the organism. Viral infection, for example, in the setting of inclusion body myositis, will have MRI features similar to polymyositis and does not form abscesses [32].

While imaging cannot definitively establish the diagnosis of primary muscle lymphoma, the imaging characteristics described above can strongly suggest this diagnosis. Lymphoma is important to suspect because it can guide the workup, limit unnecessary testing, and avoid delay in diagnosis. When intramuscular lymphoma is suspected, more generous sampling than normal of fresh tissue is often needed for pathological analysis, which may influence the strategy for obtaining biopsy [33]. Proper treatment is predicated on precise diagnosis; the current World Health Organization approach to lymphoma diagnosis is multiparametric but has become increasingly complex and typically relies on the convergence of clinical, morphologic, immunophenotypic, and genetic data. Techniques such as flow cytometry, immunophenotyping, and conventional cytogenetic analysis can only be performed on fresh [not formalin-fixed] tissue. Therefore to facilitate these studies, additional tissue may be required, which needs to be placed in sterile saline or a nutrient medium such as RPMI (Roswell Park Memorial Institute) medium to keep the cells viable.

In summary, primary and secondary muscle lymphomas are rare but present with characteristic findings on MRI and CT. The radiologist can be the first provider to suggest this diagnosis and anticipate the special biopsy preparation needed for definitive diagnosis.
